# Supplementary material for: Surface Evaluation of Orthodontic Brackets Using Texture and Fractal Dimension Analysis
Source: Materials (Basel). 2022 Mar 11;15(6):2071. doi: 10.3390/ma15062071 (PMC8951260; doi:10.3390/ma15062071)
Supplement: Supplementary file 1 [file materials-15-02071-s001.zip › materials-1589294-supplementary.pdf]

Article

Surface Evaluation of Orthodontic Brackets Using Texture and Fractal Dimension Analysis

Michał Sarul [1,*], Marcin Mikulewicz [2], Marcin Kozakiewicz [3] and Kamil Jurczyszyn [4]

Table S1. *p* Value of post hoc ANOVA results (least significant difference) for comparison of FD values between series of the same type of bracket (MS—Mini Sprint II, S—Sprint II, N—Nu-Edge, O—Orthos SS).

		**MS**		
Series	1	2	3	4
1		0.224200	0.477304	0.674151
2	0.224200		0.061807	0.108316
3	0.477304	0.061807		0.769125
4	0.674151	0.108316	0.769125	
		S		
Series	1	2	3	4
1		0.000141	0.958038	0.003099
2	0.000141		0.000160	0.199682
3	0.958038	0.000160		0.003503
4	0.003099	0.199682	0.003503	
		N		
Series	1	2	3	4
1		0.849285	0.332815	0.865850
2	0.849285		0.433121	0.983147
3	0.332815	0.433121		0.421119
4	0.865850	0.983147	0.421119	
		O		
Series	1	2	3	4
1		0.234195	0.122801	0.029355
2	0.234195		0.704622	0.275985
3	0.122801	0.704622		0.470611
4	0.029355	0.275985	0.470611	

Table S2. *p* Value of post hoc ANOVA results (least significant difference) for comparison of FD values between type of brackets (MS—Mini Sprint II, S—Sprint II, N—Nu-Edge, O—Orthos SS).

	**MS**	**S**	**N**	**O**
MS		0.000000	0.749526	0.224724
S	0.000000		0.000000	0.000016
N	0.749526	0.000000		0.369382
O	0.224724	0.000016	0.369382	
